# Is the perceived risk of fire-related injury and trust in local authorities affected by rescue services’ actual ability?

**DOI:** 10.1016/j.pmedr.2022.102030

**Published:** 2022-10-19

**Authors:** Finn Nilson, Anna Mankell

**Affiliations:** a Department of Political, Historical, Religious and Cultural Studies, Karlstad University, Karlstad, Sweden; b Centre for Societal Risk Research, Karlstad University, Karlstad, Sweden; c Center for Civil Society Research, Marie Cederschiöld University, Stockholm, Sweden

**Keywords:** Fire-related morbidity, Injury, Trust, Risk, Prevention

## Abstract

Rescue services are one of the most crucial actors in providing fire-related societal protection, particularly in terms of response times. To achieve this, considerable funding and/or full-time organisations are required – something that is not always feasible and leads to differences in rescue service related protection in different municipalities. In turn, the question is how such variations affect the perceived risk of being injured in a residential fire and levels of general institutional trust in the municipality. By utilising a dataset on risk perception and trust, combined with data on the ability and structure of municipal rescue services, this study can show that a municipal’s average perception of the risk of attaining a fire-related injury closely follows response times – indicating that individuals living in areas with less societal protection are aware of this fact. However, the results also indicate that general institutional trust and perception of risk on a municipal level seems to relate to each other in such a way that municipal types (with similar sociodemographic characteristics and rescue service ability) congregate around similar levels of perceived risk and trust. In turn, this study therefore indicates that perceived risk and trust differs in different municipalities and that efficiency measurements not necessarily encompass all these differences.

## Highlights

• Slower rescue service response times lead to an increased municipal risk perception.
• Semi-rural communities have high trust and low perceived risk.
• Semi-urban communities have low trust and high perceived risk.

## 1 Introduction

The prevalence of residential fires and the rates of fire-related mortality are well-known to fluctuate between countries (WHO, 2004), but also within countries (Jonsson et al., 2017). Often, the understanding of the variability in fire-related mortality has focused on more or less modifiable individual socio-demographic risk factors in an attempt to find solutions to solving fire-related injury inequality on both a national and regional/local level. However, although individual vulnerability – related to age, living conditions, education and employment (Ballard et al., 1992, Duncanson et al., 2002, Gilbert and Butry, 2018, Jennings, 1999, Jonsson et al., 2017, Jonsson and Jaldell, 2019, Marshall et al., 1998, Runefors et al., 2016, Xiong et al., 2015) – are crucial in understanding differences in fire mortality risk, societal protection, i.e. the municipality’s ability to protect one’s citizens, is an equally important element in explaining municipal variation in fatal fire statistics (Nilson and Bonander, 2021).

Specifically, although other actors, such as homecare staff, passers-by and neighbours can play an important role in minimising mortality risk (Runefors et al., 2021) by intervening early in the fire process, rescue services are one of the most crucial actors in providing fire-related societal protection – both in terms of preventive and reactive interventions. Rescue services, however, are prone to inefficiencies (Danielsson and Sund, 2016, Knight, 2013) and given that they are commonly organised and financed through public funding, such elements can affect the organisation of rescue services (Frantzich et al., 2019, Knight, 2013), leading to different organisational structures in different geographical areas. This organisational aspect is especially important to consider in countries with large remote areas, such as Sweden, Canada or Australia. Rural areas more often have part-time rescue services whilst urban areas have full-time forces. Although part-time organisations are less costly than full-time, the ability to respond will also be affected. Given that response times are one of the most important factors in terms of saving lives (Jaldell, 2015, Kobes and Van Den Dikkenberg, 2016, Runefors, 2020), the fact that part-time organisations (in Sweden) are on average five minutes slower (Mattsson and Juås, 1997) is noteworthy.

Apart from objectively longer response times, thereby objectively increasing the risk of fire-related deaths (Jaldell, 2015), there have been fears that part-time rescue services would affect the population’s general trust and confidence in local authorities, given that the service levels are poorer – something local Swedish policymakers also believe (Frantzich et al., 2019). Trust and confidence in local actors is important and previous studies, particularly on wildfire prevention, have shown that trust in local authorities (not merely rescue services but also healthcare, crisis response organisations, etc.) is an important aspect of improving both individual and community preparedness through a strengthened social capital (Hesseln, 2018). In turn, this may be connected to the fear that whether rescue services are manned full- or part-time could negatively affect the perception of risk amongst the population (Holmgren and Weinholt, 2016).

Although the objective fire-related risk of mortality and morbidity increases with longer response times, it is important to highlight that issues of trust and risk perception are often more closely connected to expectations and subjective experiences rather than objective truths. Median response times, regardless of organisational type, are, in most cases, within the 15 min that a large majority of the Swedish population believe (and accept) it takes for rescue services to arrive. Also, it is widely accepted that fire protection cannot be as encompassing in rural areas as in urban (Sandin and Wester, 2010). Regardless, this belief – that a lower level of service will negatively affect the confidence in societal actors and increase risk perception – is well-established in rescue service organisations (see for example (Knight, 2013)). The empirical foundation for such fears, however, is relatively unestablished. As such, this study aims to investigate how the actual and objective service levels in municipal rescue service organisations relates to a municipal’s aggregate perceived risk of attaining a fire-related injury and the level of trust in local institutions.

## 2 Material and methods

### 2.1 Material

This study uses a dataset from the project Tillitsbarometern (Trägårdh, 2019), a population survey that studies local variations in trust among a random sample of individuals aged 18–85 living in one of 49 municipalities (deliberately chosen to represent a maximum variation in factors that are assumed to affect different forms of trust, such as share of citizens with immigrant background, socioeconomic diversity, and share of reported crimes per citizen) around Sweden. The number of respondents varied in each municipality (between 180 and 2584, in total 13,667) and were randomly chosen and contacted by mail. As is common in survey studies, the sample has some overweight towards women, well-educated individuals, and people with higher incomes. The sample reflects the Swedish population well in regard to number of respondents living in rural and urban municipalities. Data was collected in the autumn of 2020 and beginning of 2021 and included several risk-related variables, including the question of how the individual perceived their own risk of being injured in a residential fire. The wording of the question, specifically asking about the risk of injury and not the risk of residential fire, is due to two perspectives. Firstly, given that the overall survey is related to the trust in others (both institutions and other people), simply asking about the perceived fire risk would not necessarily include dimensions of potentially needing help from others. Secondly, although all residential fires are unwanted, a large majority of residential fires do not lead to injury and do not require assistance from neighbours nor rescue services (Gilbert, 2021). The overarching issue at hand is to minimise injuries and fatalities due to fires and help ascertain why the fire fatality trends are no longer decreasing (Jonsson et al., 2016). As such, if one can handle a fire by oneself, the risk of fire may be considered great, but the risk of fire-related injury is considered small. Consequentially, the question focuses on injury risk.

In terms of the measure of general trust in local institutions, this was constructed by combining several questions into an index (see appendix 1 for more specific information on these questions). It is important to note that the specific question of trust in rescue services was not present in the questionnaire – meaning that the specific relationship between perceived risk, objective ability and trust in rescue services cannot be investigated. However, by using a more general measure – determined by the municipal mean value of all respondent’s responses to questions regarding their trust in the ruling party/parties in the municipality, the preschools in the municipality, the elementary schools in the municipality, the local police force, elderly care in the municipality, the local employment agency, and the local healthcare – the relationship between a municipality’s rescue service ability and the mean general institutional trust and perceived risk can be presented. The response scale for all questions was a five-step Likert scale, ranging from “very low or none” (1) to “very high” (5).

Finally, data regarding average municipal response times and funding of rescue services was collected from the publicly available database Kolada, as was other municipal data that was relevant from a fire morbidity/mortality risk perspective such as population density, GRP (regional GNP) per capita, percentage of the population with tertiary education, percentage of the population who are smokers, etc. Data regarding which organisational solutions for rescue services that different municipalities had was retrieved from the Swedish Civil Contingency Agency who collect and distribute such data. In Sweden, the local municipality finances rescue services. Within each municipality there are often several fire stations that are organised in three different overarching categories: full-time, semi part-time, or fully part-time. In terms of the two versions of part-time, the differences are related to whether all part-time personnel need to rendezvous at the fire station (fully part-time) or whether one individual is particularly prepared, with an emergency vehicle at home, and able to go straight to the incident whilst remaining personnel comes afterwards (semi part-time). In each municipality different combinations of full-time and part-time organisations exist. To compare municipalities, each municipality was categorised as either “majority full-time”, i.e., municipalities where most stations were staffed full-time, “partly full-time”, i.e., there was at least one full-time station though a majority were part-time versions, and “fully part-time”, i.e., municipalities with no full-time station.

### 2.2 Methods

The statistical analysis of this paper was conducted in four steps. Firstly, the municipal mean value of general trust and perceived risk of being injured in a residential fire was calculated. Secondly, using these mean values the analysis departs from a two-sided Pearson correlation analysis of the relationships between institutional trust, perceived risk and two variables measuring characteristics of rescue services at the municipal level, namely response time and spending. Thirdly, to understand nuances of the correlations between municipalities, a categorisation of municipalities was performed. The categorisation resulted in four groups of municipalities, constructed from their deviation from the mean value of both perceived risk and institutional trust. Fourthly, to understand the characteristics of these groups an ANOVA analysis was performed, comparing means of variables measuring both rescue service characteristics as well as mean sociodemographic characteristics of the municipalities. To conclude the statistical significance of the variations, a post-hoc test (Tukey’s HSD test) was also performed. All tests were performed using the programme SPSS (version 28.0.1).

## 3 Results

The aggregate means of perceived risk and general trust in local institutions varied in the studied municipalities. In terms of the national mean, the perceived risk (on a 0–5 Likert scale) was 2.01 (std dev 0.11) whilst the mean general trust in local institutions (also a 0–5 Likert scale) was 3.33 (std dev 0.14). When comparing our 49 municipalities, the mean perceived risk varied between 1.79 (std dev 0.8) and 2.22 (std dev 0.8) whilst the mean general trust in local institutions varied between 3.14 (std dev 0.7) and 3.67 (std dev 0.66) (appendix 2).

To start, correlations between aggregate municipal response times and spending (i.e., objective protection measures), aggregate municipal risk perception and aggregate municipal institutional trust was tested. As these comparisons were on the municipal level, n = 49. As seen in Table 1, strong and significant correlations are seen between the aggregate perceived risk and the two objective measures response time and funding per capita. However, whilst the relationship between perceived risk and response times was in line with expectations, i.e., that the perceived risk increases with longer response times, the relationship between institutional trust and response times showed, surprisingly, that trust was largely unaffected with a slight tendency towards an increase with longer response times (this result was only significant at the 0.1 level). In terms of funding per capita, perceived risk increased with increased funding.

**Table 1:** Correlation analysis, Pearson. (**= sig. at 0.01, *= sig at 0.05).

	Institutional trust	Perceived risk	Response time	Spending per capita
Institutional trust	1	−0.169	0.248	−0.141
Perceived risk		1	0.539**	0.310*
Response time			1	0.221
Spending per capita				1

**Fig. 1:**
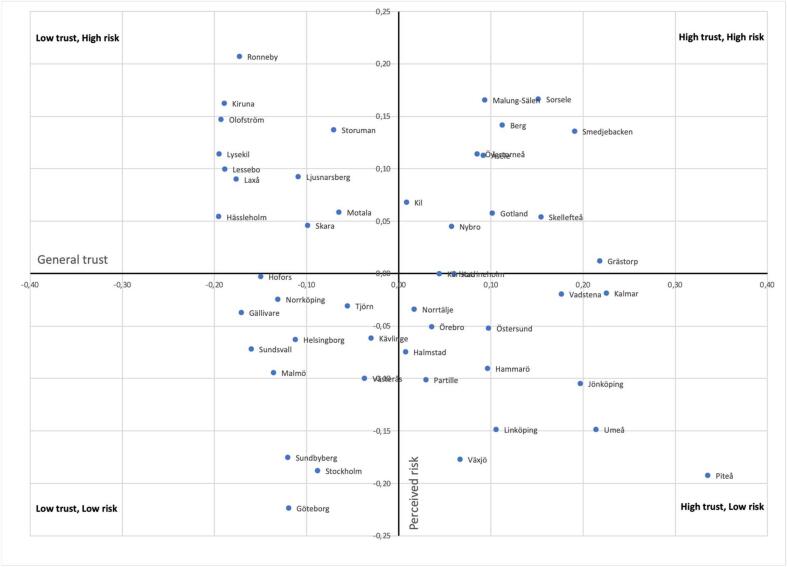
The relationship between the municipal aggregated rate of perceived risk and trust in local institutions expressed as the deviation from the mean.

Given the large geographical variations within a country like Sweden, the question arises whether there are nuances to these relationships. To discover such nuances, an index was created based on the aggregate municipal score on trust and the aggregate municipal score on perceived risk, both based on the municipal’s deviation from the mean. The resulting scatterplot is seen in Fig. 1. Given that the scale is the municipalities deviation from the mean, four groups of municipalities of relatively equal size are created. As seen in Fig. 1, the municipalities can have either a higher or lower perceived risk than average, and a higher or lower level of institutional trust than average. From this scatterplot, an interesting pattern of municipality types emerges, where municipalities are clustered along an urban/rural dimension, where for instance the top right quadrant represent rural municipalities, and the bottom right quadrant represent mainly urban municipalities.

Using this index, the four groups’ averages in relation to rescue service characteristics can be compared. As seen in Table 2, the four groups differ considerably. Starting with the high trust/high perceived risk (HTHR) group, i.e., the top-right group in Fig. 1, this group has significantly longer response times and higher costs per capita than the other groups. Interestingly, the low trust/low perceived risk (LTLR) group, that follows the same positive correlation principle as HTHR, is at the opposite end of the spectra with the quickest response times and lowest costs. The LTHR and HTLR groups, meanwhile, position themselves in-between the above-mentioned groups in terms of both costs and response times. Overall, the results seem to indicate that there is a connection between the municipalities’ aggregate perceived risk and their actual risk (based on response times being higher in high-risk groups). As also seen in Table 2 the type of rescue service organisation also varied in the different groups – with a greater percentage majority full-time in the LTLR group whilst the HTHR group has a greater percentage of fully part-time. Between the LTHR and HTLR groups, a greater percentage of municipalities were fully part-time in the LTHR group.

**Table 2:** Characteristics of the HTHR, LTHR, HTLR and LTLR groups, mean comparisons and percentages of group.

		HTHR		LTHR		HTLR		LTLR	
		Mean	Std. Dev.	Mean	Std. Dev.	Mean	Std. Dev.	Mean	Std. Dev.
Rescue Service data									
	Response time (min)	14.65	4.15	12.48	2.93	11.08	1.20	10.29	1.42
	Spending (SEK/capita)	1484.54	834.50	1236.39	328.10	814.27	249.03	753.17	392.24

Municipality data									
	dependency ratio	0.93	0.10	0.92	0.08	0.80	0.10	0.72	0.14
	age 65+ and living alone (%)	43.75	2.73	43.25	2.25	42.74	2.61	46.75	5.23
	tertiary education (25–65 years) (%)	33.16	6.75	30.99	4.04	46.87	7.36	48.90	11.12
	share smokers (%)	6.56	2.05	7.96	1.84	6.25	1.71	6.75	1.34
	population density	2.08	1.56	2.81	1.42	4.26	0.94	6.02	2.40
	Gross Regional Product (GRP)/capita	281.34	78.59	332.56	140.63	346.43	57.35	536.69	229.0

Rescue Service Org.	% of group								
	Mainly fulltime	0		0		0		33	
	Partly fulltime	54		54		84		50	
	Mainly parttime	46		46		16		17	

Socio-geography									
	Urban	0		0		8		42	
	Semi-urban	31		36		61		42	
	Rural	69		64		31		16	

Although it is clear that the four groups differ considerably in terms of rescue service-related variables, as is also seen in Table 2, in terms of other fire-relevant municipal sociodemographic characteristics, there are also a large number of statistical differences with Tukey’s test showing that the mean values of all included variables differed significantly between the four groups. Summarised, the four groups’ general characteristics can be described as follows;

- HTHR – rural municipalities with low GRP per capita, high rescue service costs and slow response times. Rescue services are organised in part-time structures. The municipalities have a high dependency ratio and large share of older adults living alone. These municipalities have the lowest GRP/capita of all four groups.
- LTHR – semi-rural municipalities, i.e., smaller rural municipalities where many commute to nearby municipalities with slightly larger towns. Rescue service costs are high and response times slower. Rescue services are typically organised as part-time structures though full-time does also exist. The municipalities have, much like the HTHR group, high dependency ratios and a large share of elderly living alone but with a higher population density than the HTHR group. Compared to the more rural HTHR group, the municipalities in this group have fewer inhabitants with tertiary education and have the largest percentage of smokers.
- HTLR – semi-urban municipalities, i.e., municipalities with larger towns (over 50,000 inhabitants) but also with more rural areas. Rescue service organisations are therefore predominantly full-time but part-time stations are also common. Costs on rescue services are higher than in LRLT and response times slower. The municipalities have a higher dependency ratio than the LRLT group and a smaller share of older adults living alone. Furthermore, this group has substantially lower GDPR/capita than the LTLR group.
- LTLR – highly urban municipalities with professional full-time firefighter organisations resulting in low costs and fast response times. The municipalities have low dependency ratios, high education levels, but also the highest share of older adults living alone. This group has the highest GRP/capita of all four groups.

## 4 Discussion

This study shows several aspects that are critical in understanding municipal variations in fire-related risk perception, municipal rescue service’s actual ability and trust in local institutions. On a national level, comparing aggregate municipal data, the data suggests that increases in response times is correlated with an increase in the municipal aggregate perceived risk of being injured in a fire. Also, the aggregate level of general trust seems to be higher in municipalities with higher rescue service funding, slower response times and more part-time organisations.

Starting with perceived risk and correlation analysis, the objective measurement of protection, response times, is closely correlated to how high individuals on average in a municipality rate their risk of being injured in a fire. Given the importance of time in saving lives (Jaldell, 2015, Kobes and Van Den Dikkenberg, 2016, Runefors, 2020), this correlation is understandable and largely rational. These results also echo findings from previous studies that have indicated that Swedes in general are well aware, and acceptant, of rural areas having a poorer level of service in terms of response times and that individuals living in rural areas have a greater responsibility to protect themselves from residential fires (Sandin and Wester, 2010).

The division of municipalities into four different groups, defined in relation to their deviation from the mean, clarifies this relationship further. Specifically, based purely on the average trust/risk deviation from the mean, a highly uniformly division into a rural, semi-rural, semi-urban and urban group was revealed. Clearly, it would seem that – in similarity to the overall correlations – the perceived risk of being injured in a residential fire follows urbanisation, with rural municipalities scoring a higher average level of perceived risk to urban municipalities. However, in terms of the municipal mean score of trust, no such obvious explanations can be seen. Rather, lower average levels of trust are seen in highly urban municipalities and semi-rural municipalities. Also, very few similarities in terms of rescue service organisation, demographics, rescue service ability, etc., are seen, thereby indicating that there may be other factors that affect trust that were not included in this study.

A commonly-held fear from policymakers is that changing from full-time to part-time organisations would negatively affect confidence and trust (Knight, 2013) or perceived risk (Holmgren and Weinholt, 2016), as a result of a reduction in service. Clearly, although no causal relationship can be determined, this study shows that risk perception is lower when service levels are poorer. However, in terms of general trust this seems to be less affected by the type of organisation or response times. The fact that trust seems to be less affected by objective service levels can be understood in several ways. First, due to part-time firefighters having two occupations and therefore a natural presence in the community, they are tightly intertwined in the local community and contribute with considerable social capital (Almklov et al., 2018). As such, although the actual and objective level of service is poorer with part-time organisations in HTHR municipalities, the general level of trust may still be high. Secondly, trust is heavily embedded in expectations.As discussed previously, there is a strong societal acceptance that urban areas will have quicker response times than rural areas (Sandin and Wester, 2010). As such, individuals living in very rural municipalities will not expect high levels of service. Whilst individuals living in very urban areas will expect high levels of service, urban areas in Sweden have had considerable problems related to rescue services and socio-economically weak areas (Hallin et al., 2010). However, this does not explain why trust fails to follow a similar urban-rural pattern as seen in terms of risk perception. It could be hypothesised that a division of municipalities into smaller areas – thereby creating urban and rural areas within the semi-rural and semi-urban municipalities – could show similar patterns as seen when purely focusing on very urban or very rural municipalities. However, response times are not available for smaller geographical areas as there are too few callouts. Instead, qualitative studies or case studies on particular municipalities may provide more definitive answers.

The semi-urban communities, that to some extent have an ideal combination of high trust and low perceived risk, may have touched upon a sweet spot of sufficient presence in the community though simultaneously accomplishing quick response times by combining full- and part-time organisations. Alongside the relatively high societal protection, the individual vulnerability in these municipalities is low – therefore reiterating the fact that both these elements are as important (Nilson and Bonander, 2021). Previous Swedish studies have shown that living alone is a considerable risk factor (Nilson et al., 2020), that  tertiary education is a protective factor (Jonsson and Jaldell, 2019) and that smoking is a considerable risk factor (Jonsson et al., 2017). The semi-urban (HTLR) group has the lowest percentage of smokers, the lowest percentage of older adults living alone and amongst the highest percentage of individuals with tertiary education.

A crucial factor in terms of both trust and perceived risk is the issue of expectations. As discussed previously, there is a strong societal acceptance that urban areas will have quicker response times than rural areas (Sandin and Wester, 2010). As such, individuals living in very rural municipalities will not expect high levels of service, whilst individuals living in very urban areas will. It could be hypothesised that the same is true within the semi-rural and semi-urban municipalities. In these municipalities there will often be areas that are urban and areas that are rural (with more rural areas in the semi-rural and more urban areas in the semi-urban). To study this, smaller divisions are needed. However, response times are not available for smaller geographical areas as there are too few callouts and therefore individual’s expectations need to be studied in a different manner, as would longitudinal changes in organisational type. Likewise, although the quantitative differences in risk perception and trust shown in this paper clearly indicate that they are affected by the capabilities of rescue services, how this relationship is understood needs to be studied further to utilise the results. For example, qualitative studies or case studies on municipalities that have made changes in their financing or organising of rescue services may provide more definitive answers.

There are some limitations to this study that need to be raised. First, this study focuses on the role of rescue services in terms of the objective societal protection against fire-related morbidity and mortality. Whilst rescue services are the main provider, this perspective could be seen as somewhat crude. Fire protection is, ideally, a holistically constructed endeavour with many different actors involved in the prevention of, and reaction to, fires. Not least, private security companies and healthcare deliverers act as first-responders in many Swedish municipalities. In practice, given that response times are assessed in terms of the arrival of rescue services, response times may be lower than official reports suggest (Mojir and Pilemalm, 2016). However, although this is true, such programmes have been shown to have a relatively low effect on the risk of death (Sund and Jaldell, 2018). Whilst this does not eliminate the potential gains of such programmes, given other value-adding elements (Weinholt and Andersson Granberg, 2015) or the fact that rescues are performed by healthcare deliverers (Runefors et al., 2021), for the sake of actual effect on injury it seems as if such programmes have such a limited effect that they can be disregarded. Also, such interventions are found in most municipalities meaning that it is unlikely that there are any major effects in the difference between municipalities. Secondly, there are some issues with the data collection. Questionnaires almost always have a certain selection bias in terms of which groups answer. Whilst attempts have been made to minimise this through detailed analysis of non-responses, one must view the results thereafter. Similarly, the data collection is not national. Instead, the survey used a purposeful selection of municipalities that were deemed representative in terms of the Swedish population’s differences in trust. As such, we believe that the results most likely represent the Swedish population although this cannot be entirely certain. Lastly, and perhaps most importantly, whilst a majority of the variables studied in this paper refer directly to rescue services, the trust variable relates to a general trust of local institutions. In practice this means that the trust measure is an average of different elements of trust – including trust in healthcare, schools, politicians, the police force, etc. (see appendix 1). Clearly it would have been preferable to have a specific measure of trust in rescue services given that an individual may have a high level in trust in certain municipal services whilst very low in other services. However, this is generally not the case. Rather, the variance in the trust in different local institutions is low (Christensen and Lægreid, 2005) and to our knowledge there is no evidence to suggest that there would be a greater variance in terms of rescue services than in relation to other municipal services. Also, as highlighted previously, local fire prevention cannot solely be attributed to rescue services. Healthcare providers and police are active actors in fire prevention and local politicians are central actors in determining funding and resources. As such, whilst not a perfect measure, we believe that using general local institutional trust as a measure is a sound alternative to a specific measure on trust in rescue services. However, the results in this paper should be viewed with this in mind and in future data collections specific questions regarding trust in rescue services should be included.

## 5 Conclusions

The abilities of rescue services, particularly in terms of response times, have been shown to be critical in saving lives. Simultaneously, the abilities of rescue services are hampered by geographical challenges and financial constraints meaning that response times can vary considerably. This study can show that when comparing municipalities, the average perception of the risk of attaining a fire-related injury closely follows response times – indicating that individuals living in areas with lower societal protection are aware of this fact. However, the results also indicate that the ability of rescue services does not, on average, affect general trust in the local community in the same way. Rather, this study indicates that rescue service ability and organisation affects trust in different ways for different types of municipalities, and that general trust can be both high despite low levels of service and low despite high levels of service. Although further studies are needed, the results highlight an important issue in the relationship between the ability of local authorities, individual’s trust in these authorities, and individual’s perceived risk.

## Declaration of Competing Interest

The authors declare that they have no known competing financial interests or personal relationships that could have appeared to influence the work reported in this paper.

## Data Availability

Data will be made available on request.
